# Redox-Regulated Magnetic Conversions between Ferro- and Antiferromagnetism in Organic Nitroxide Diradicals

**DOI:** 10.3390/molecules28176232

**Published:** 2023-08-24

**Authors:** Fengying Zhang, Zijun Zhang, Yali Zhao, Chao Du, Yong Li, Jiaqi Gao, Xiaobo Ren, Teng Ma, Boqiong Li, Yuxiang Bu

**Affiliations:** 1 Department of Materials Science and Engineering, Jinzhong University, Jinzhong 030619, China; zx88dc@sina.com (C.D.); lytyut@126.com (Y.L.); gjq5207@outlook.com (J.G.); rxb@jzxy.edu.cn (X.R.); mateng1022@tyust.edu.cn (T.M.); lbq@jzxy.edu.cn (B.L.); 2 Shanxi Province Collaborative Innovation Center for Light Materials Modification and Application, Jinzhong 030619, China; 3 Department of Chemistry and Chemical Engineering, Jinzhong University, Jinzhong 030619, China; 18334858404@163.com; 4 School of Chemistry and Chemical Engineering, Shandong University, Jinan 250100, China; byx@sdu.edu.cn

**Keywords:** magnetic modulation, redox-active couplers, nitroxide diradical, ferromagnet, antiferromagnet, DFT calculation

## Abstract

Redox-induced magnetic transformation in organic diradicals is an appealing phenomenon. In this study, we theoretically designed twelve couples of diradicals in which two nitroxide (NO) radical groups are connected to the redox-active couplers including p-benzoquinonyl, 1,4-naphthoquinyl, 9,10-anthraquinonyl, naphthacene-5,12-dione, pentacene-6,13-dione, hexacene-6,15-dione, pyrazinyl, quinoxalinyl, phenazinyl, 5,12-diazanaphthacene, 6,13-diazapentacene, and 6,15-diazahexacene. As evidenced at both the B3LYP and M06-2X levels of theory, the calculations reveal that the magnetic reversal can take place from ferromagnetism to antiferromagnetism, or vice versa, by means of redox method in these designed organic magnetic molecules. It was observed that p-benzoquinonyl, 1,4-naphthoquinyl, 9,10-anthraquinonyl, naphthacene-5,12-dione, pentacene-6,13-dione, and hexacene-6,15-dione-bridged NO diradicals produce antiferromagnetism while their dihydrogenated counterparts exhibit ferromagnetism. Similarly, pyrazinyl, quinoxalinyl, phenazinyl, 5,12-diazanaphthacene, 6,13-diazapentacene, and 6,15-diazahexacene-bridged NO diradicals present ferromagnetism while their dihydrogenated counterparts show antiferromagnetism. The differences in the magnetic behaviors and magnetic magnitudes of each of the twelve couples of diradicals could be attributed to their distinctly different spin-interacting pathways. It was found that the nature of the coupler and the length of the coupling path are important factors in controlling the magnitude of the magnetic exchange coupling constant *J*. Specifically, smaller HOMO-LUMO (HOMO: highest occupied molecular orbital, LUMO: lowest unoccupied molecular orbital) gaps of the couplers and shorter coupler lengths, as well as shorter linking bond lengths, can attain stronger magnetic interactions. In addition, a diradical with an extensively π-conjugated structure is beneficial to spin transport and can effectively promote magnetic coupling, yielding a large |*J*| accordingly. That is, a larger spin polarization can give rise to a stronger magnetic interaction. The sign of *J* for these studied diradicals can be predicted from the spin alternation rule, the shape of the singly occupied molecular orbitals (SOMOs), and the SOMO-SOMO energy gaps of the triplet state. This study paves the way for the rational design of magnetic molecular switches.

## 1. Introduction

Organic magnetic materials have received considerable attention due to their advantages, which include being lightweight, environmentally friendly, and simple to manufacture, as well as their potential applications in the fields of optics, electricity, and magnetism [1,2,3,4,5,6]. A diradical is the most elementary magnetic molecule or molecular magnet and is the basis of high-spin-state molecular materials. Diradicals have become a focus of materials science recently. For a diradical, when two unpaired electrons occupy nearly degenerate spatial orbitals, a parallel-spin orientation gives rise to a triplet ground state (ferromagnetic coupling) while an antiparallel-spin orientation leads to a singlet ground state (antiferromagnetic coupling) [7]. A common type of organic diradical is composed of two single radical groups as the spin sources bridging through a coupler, which has been extensively investigated experimentally and theoretically [8,9,10,11,12,13,14,15,16,17,18]. The magnetic coupling constant *J* is largely dependent on the interaction between two spin sources. The larger the |*J*| value is, the stronger the magnetic coupling interaction between two spin sources is. Positive and negative *J* values represent the ferromagnetic and antiferromagnetic interactions between two spin sources, respectively.

Organic diradicals with switchable spin states or magnetic properties have a wide range of applications in organic spintronics, molecular electronics, data storage devices, and so on [19,20,21]. It is reported that magnetic reversal phenomena for organic diradical system can be realized using a variety of methods, including torsional effect [21,22]; chemical doping [1,23]; proton [24,25,26], temperature [27], and redox induction [23,28,29,30]; and photo-induced photochromics [31,32,33]. Among these methods, redox-modulated magnetic switching is attractive and likely to find extensive technological applications in the field of magnetic materials. For example, Ali and coworkers [28] demonstrated that a redox reaction can effectively regulate the magnetic properties of diradicals from ferro- and antiferromagnetism, in which two conformationally restricted nitroxide (NO) radical groups are attached to the meta-phenylen coupler with redox activity. In addition, Song et al. [30] explored the magnetic coupling characteristics of the core-modified porphyrin-bridged verdazyl, nitronyl nitroxide, and imino nitroxide diradicals and found that the magnitude and sign of their magnetic coupling constants *J* can be changed through a redox reaction. Furthermore, we discussed diaza effects on magnetic coupling characteristics in diaza-benzo[k]tetraphene-bridged NO-based diradicals and noticed that further dielectron oxidation can significantly change the magnitude or sign of *J* [23]. Overall, for organic diradicals, the key to magnetic regulation through redox induction is to find coupling units with redox property.

**Scheme 1:**
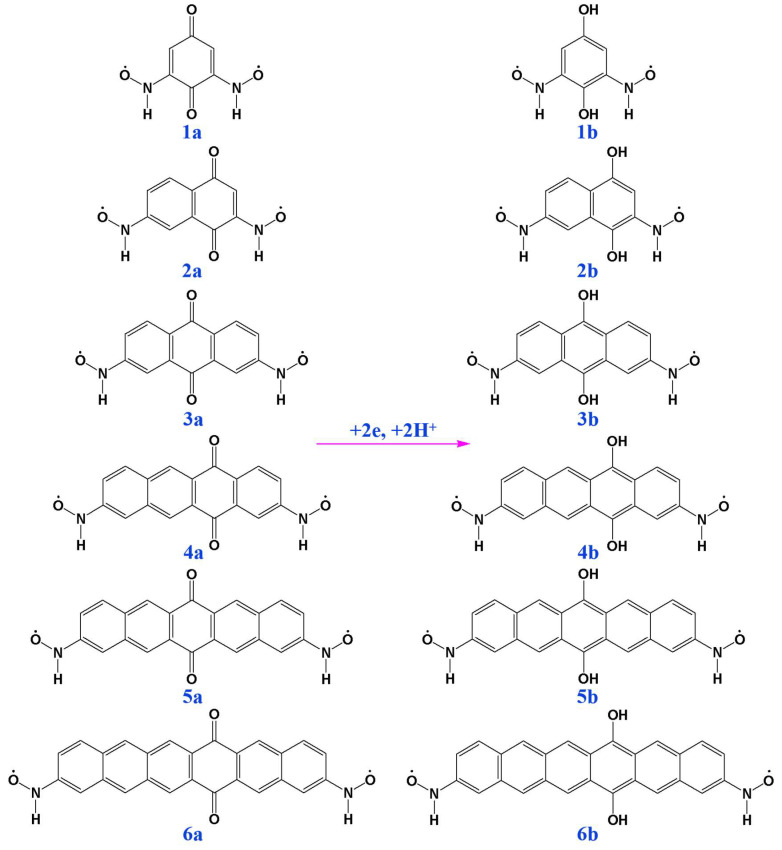

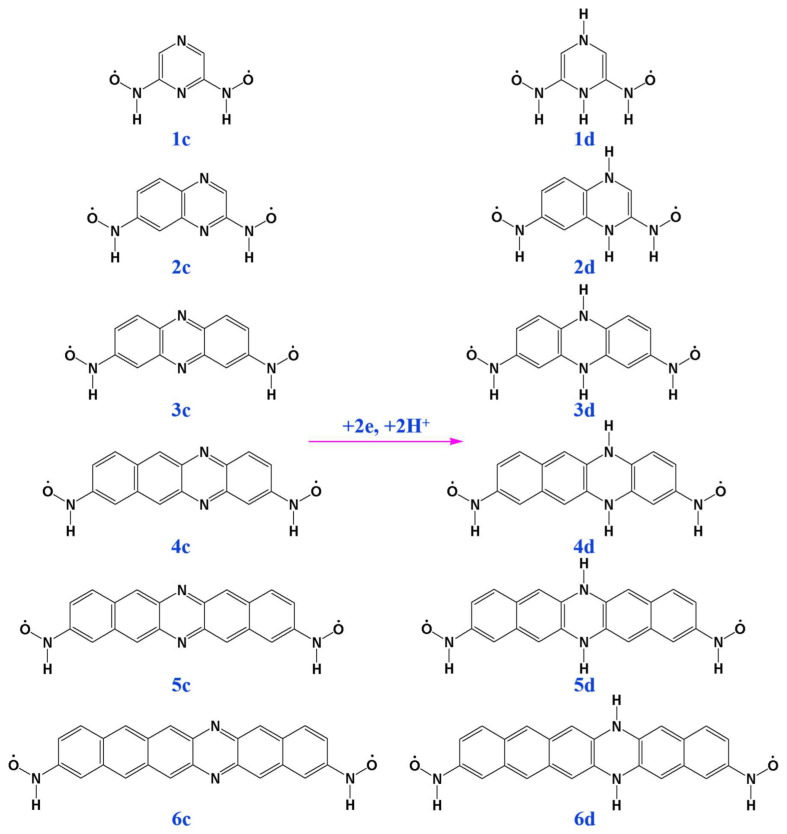
Schematic diagram of the undihydrogenated and dihydrogenated diradicals with two nitroxide (NO) radical groups. The redox-active coupling units are p-benzoquinone, 1,4-naphthoquinone, 9,10-anthraquinone, naphthacene-5,12-dione, pentacene-6,13-dione, hexacene-6,15-dione, pyrazine, quinoxaline, phenazine, 5,12-diazanaphthacene, 6,13-diazapentacene, and 6,15-diazahexacene and their dihydrogenated counterparts. They are denoted as **1a**, **1b**, **2a**, **2b**, **3a**, **3b**, **4a**, **4b**, **5a**, **5b**, **6a**, **6b**, **1c**, **1d**, **2c**, **2d**, **3c**, **3d**, **4c**, **4d**, **5c**, **5d**, **6c**, and **6d**, respectively.

Inspired by the abovementioned research findings, in this study, we chose p-benzoquinone, 1,4-naphthoquinone, 9,10-anthraquinone, naphthacene-5,12-dione, pentacene-6,13-dione, and hexacene-6,15-dione as couplers with redox activity and the relatively stable NO radicals as spin centers to construct six novel diradicals, that is, **1a**, **2a**, **3a**, **4a**, **5a**, and **6a**, as illustrated in Scheme 1. By undergoing a two-electron-two-proton (2e-2H^+^) reduction (or dihydrogenation) process, **1a**, **2a**, **3a**, **4a**, **5a,** and **6a** can convert to **1b**, **2b**, **3b**, **4b**, **5b**, and **6b**, respectively. In addition, we also selected pyrazine, quinoxaline, phenazine, 5,12-diazanaphthacene, 6,13-diazapentacene, and 6,15-diazahexacene as coupling units with redox peculiarity and the NO radicals as spin centers to construct six other novel diradicals, that is, **1c**, **2c**, **3c**, **4c**, **5c**, and **6c**, as displayed in Scheme 1. Clearly, **1c**, **2c**, **3c**, **4c**, **5c**, and **6c** could undergo a 2e-2H^+^ process to convert to **1d**, **2d**, **3d**, **4d**, **5d**, and **6d**, respectively. The calculated results indicate that magnetic conversions between ferromagnetic and antiferromagnetic exchange coupling take place for twelve couples of designed diradicals (**1a ↔ 1b**, **2a ↔ 2b**, **3a ↔ 3b**, **4a ↔ 4b**, **5a ↔ 5b**, **6a ↔ 6b**, **1c ↔ 1d**, **2c ↔ 2d**, **3c ↔ 3d**, **4c ↔ 4d**, **5c ↔ 5d**, and **6c ↔ 6d**) upon reduction or oxidization. We found that the differences in the magnetic behaviors and magnetic magnitudes of each of the twelve couples of diradicals could be attributed to their distinctly different spin-interacting pathways [24,28,29,30]. In particular, the nature of the coupler and the length of the coupling path are critical factors in determining the magnetic magnitude of these diradicals. More precisely, smaller HOMO-LUMO (HOMO: highest occupied molecular orbital, LUMO: lowest unoccupied molecular orbital) gaps of the couplers [23,34,35] and shorter coupler lengths, together with shorter linking bond lengths, can obtain stronger magnetic interactions [13]. Furthermore, a diradical with an extensively π-conjugated structure is conducive to spin transport and can effectively facilitate magnetic coupling, generating a large |*J*| accordingly [13]. In other words, a larger spin polarization can give rise to a stronger magnetic interaction. The observed magnetic switching phenomena and the nature of the corresponding ground states of these diradical molecules can be accounted for using the spin alternation rule [36,37], the singly occupied molecular orbital (SOMO) effect [38], and the SOMO-SOMO energy gaps in the triplet states. Furthermore, another spin center, the tert-butyl nitroxide radical, is also considered to verify the conclusion of the magnetic switching induced by the redox induction for **2a**, **2b**, **3a**, **3b**, **2c**, **2d**, **3c**, and **3d** (namely **2a′**, **2b′**, **3a′**, **3b′**, **2c′**, **2d′**, **3c′**, and **3d′**). It is worth mentioning that nitroxide-based diradicals have been extensively studied both experimentally and theoretically to date [9,10,13,22,23,24,25,28,29,30,39,40,41]. This work provides a very useful theoretical basis for the synthesis of nitroxide-based diradicals and offers new insights into the rational design of magnetic switchable diradicals for further applications.

## 2. Results and Discussion

### 2.1. Magnetic Couplings and Molecular Geometries

**Figure 1:**
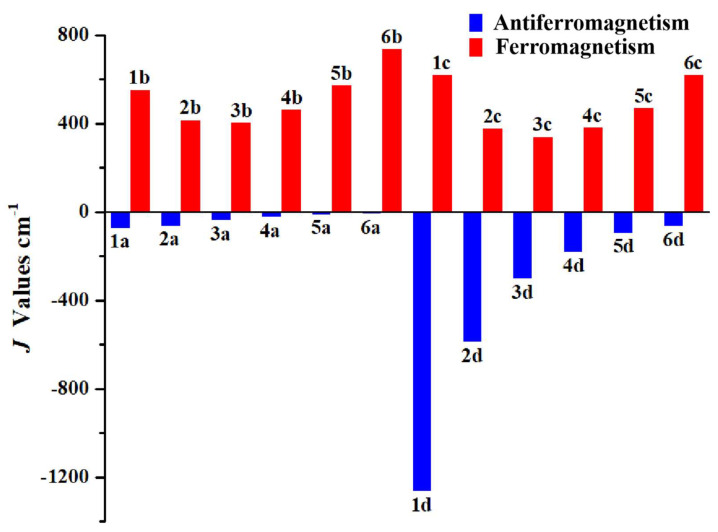
Histogram of magnetic coupling constants (*J*, in cm^−1^) of **1a**, **1b**, **2a**, **2b**, **3a**, **3b**, **4a**, **4b**, **5a**, **5b**, **6a**, **6b**, **1c**, **1d**, **2c**, **2d**, **3c**, **3d**, **4c**, **4d**, **5c**, **5d**, **6c**, and **6d**. The blue bars denote the diradicals with antiferromagnetism while the red bars denote the diradicals with ferromagnetism.

The redox-induced magnetic reversal in pure organic diradical systems is exceedingly attractive. In this section, we discuss in detail the redox-induced magnetic transformation for twelve couples of NO-based diradicals (**1a ↔ 1b**, **2a ↔ 2b**, **3a ↔ 3b**, **4a ↔ 4b**, **5a ↔ 5b**, **6a ↔ 6b**, **1c ↔ 1d**, **2c ↔ 2d**, **3c ↔ 3d**, **4c ↔ 4d**, **5c ↔ 5d**, and **6c ↔ 6d**). The detailed data including the energies of the closed-shell singlet (CS), broken-symmetry (BS) open-shell singlet, and triplet (T) states; the <S^2^> values; the energy gaps between the pure singlet state and triplet state (Δ*E*_ST_); and the spin coupling constants *J* of all studied diradicals calculated at the B3LYP/6-311++G(d,p) level of theory are presented in Table S1. Additionally, the *J* values estimated at the UM06-2X/6-311++G(d,p) level are also shown in Table S2 for the purpose of comparison. The results indicate that the calculated *J* values are relatively accurate by adopting B3LYP and M06-2X functionals, and the magnetic conversion through the redox modulation method occurs in these twelve couples of diradicals. Compared with the M06-2X functional, the B3LYP functional tends to overestimate *J* [23,29,35]. In the forthcoming discussion, all data are derived from the B3LYP functional. As displayed in Figure 1, it can be seen that the diradicals **1a**, **2a**, **3a**, **4a**, **5a**, and **6a** produce antiferromagnetism, while their dihydrogenated counterparts **1b**, **2b**, **3b**, **4b**, **5b**, and **6b** exhibit ferromagnetism. Analogously, the diradicals **1c**, **2c**, **3c**, **4c**, **5c**, and **6c** present ferromagnetism, while their dihydrogenated counterparts **1d**, **2d**, **3d**, **4d**, **5d**, and **6d** show antiferromagnetism. Moreover, we notice that the estimated |*J*| values for the diradicals with antiferromagnetism decrease gradually with an extension of the coupling path between two spin centers, while the *J* values of the diradicals with ferromagnetism decrease first and then increase. Specifically, the |*J*| values follow the order **1a** > **2a** > **3a** > **4a** > **5a** > **6a** and **1d** > **2d** > **3d** > **4d** > **5d** > **6d**, whereas the *J* values are in the orders **1b** > **2b** > **3b** < **4b** < **5b** < **6b** and **1c** > **2c** > **3c** < **4c** < **5c** < **6c**. The structural and electronic properties of the coupler associated with the geometric character of a specific diradical influence the effective magnetic coupling between two spin centers. It should be noted that Table S3 presents the *J* values calculated for the other four couples of bis(tert-butyl)-nitroxide-based diradicals (**2a′ ↔ 2b′**, **3a′ ↔ 3b′**, **2c′ ↔ 2d′**, **3c′ ↔ 3d′**) at the B3LYP/6-311++G(d,p) level. It is further proved that redox induction can indeed modulate the magnetic transformation. The magnitudes of *J* for these bis(tert-butyl)-nitroxide-based diradicals are basically comparable to that of the corresponding nitroxide-based ones except for **2b′**. This deviation is due to the poor planarity of **2b′**.

For a π-conjugated diradical, the magnetic exchange coupling between two spin centers is mainly through the bond. The shortest coupling path can be roughly expressed as the sum of the bond lengths of the adjacent atoms between two spin centers. All twelve studied couples of diradicals are π-conjugated. The bond lengths of the adjacent atoms between two spin centers of the twelve couples of diradicals through the shortest coupling path are shown in Figure S1. For **1a**, **2a**, **3a**, **4a**, **5a**, and **6a**, the shortest coupling path between two spin centers gradually becomes longer with an increase of the two carbon–carbon bonds in the benzene ring of the coupler, which is not conducive to spin coupling, and, correspondingly, the magnetic interaction successively weakens. A similar case occurs for **1d**, **2d**, **3d**, **4d**, **5d**, and **6d**. This is in good conformity with the idea that shorter coupler lengths attain stronger magnetic interactions [13]. For **1b**, **2b**, **3b**, **4b**, **5b**, and **6b**, both the coupler length and the HOMO-LUMO gap of the coupler are crucial in determining the magnetic coupling. In comparison with **1b** (*J* = 551.6 cm^−1^), the longer coupler lengths of **2b** (*J* = 417.2 cm^−1^) and **3b** (*J* = 408.0 cm^−1^) weaken their magnetic couplings gradually. Compared with **3b**, the smaller HOMO-LUMO gaps of the couplers for **4b**, **5b**, and **6b** enhance their magnetic interactions successively; their corresponding HOMO-LUMO gaps are 3.13, 2.47, 1.97, and 1.61 eV, respectively (Table S4). A similar situation happens for **1c**, **2c**, **3c**, **4c**, **5c**, and **6c**. That is, the shorter coupler lengths of **1c** and **2c** promote their magnetic interactions, which is in contrast with **1c**. The decreasing HOMO-LUMO gaps of **3b**, **4b**, **5b**, and **6b** strengthen magnetic couplings. These results are more consistent with the view that the small HOMO-LUMO gap of the coupler can efficiently enhance the spin coupling interaction [23,34,35]. The HOMO and LUMO energy levels, as well as the HOMO-LUMO gaps of the couplers for **1b**, **2b**, **3b**, **4b**, **5b**, **6b**, **1c**, **2c**, **3c**, **4c**, **5c**, and **6c**, are shown in Table S4. Furthermore, the magnetic coupling strengths of all studied diradicals are also closely related to their structural properties.

**Figure 2:**
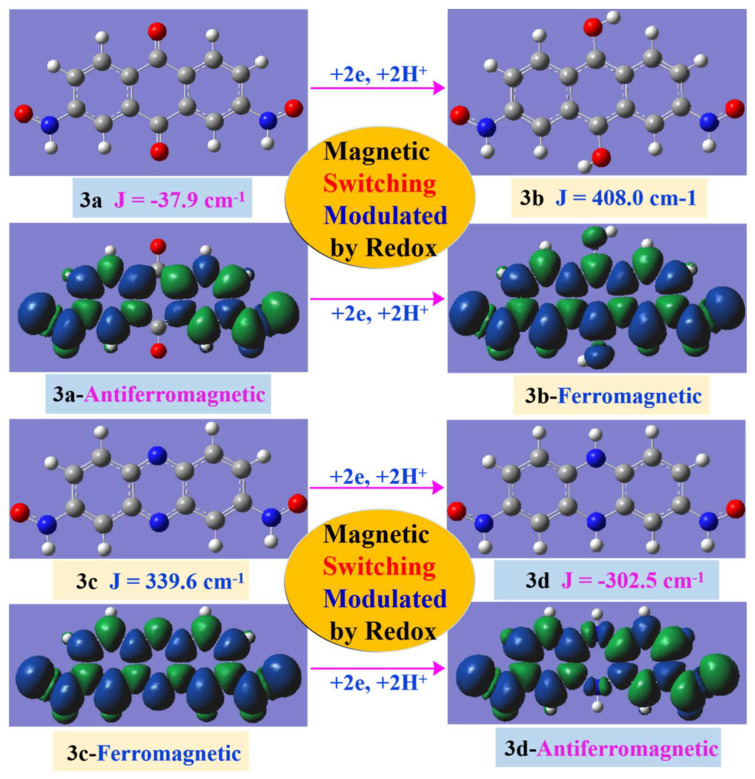
Schematic diagram of redox−modulated conversions of structures for the nitroxide−based diradicals. The figure shows the optimized geometries and spin density map for the ground states of two couples of diradicals, **3a ↔ 3b** and **3c ↔ 3d**.

The geometric optimizations in Figure 2 and Figure S1 indicate that the coupler and two NO radical groups of **1a**, **2a**, **3a**, **4a**, **5a**, **6a**, **1c**, **2c**, **3c**, **4c**, **5c**, and **6c** are coplanar while the dihydrogenated counterparts of **1b**, **2b**, **3b**, **4b**, **5b**, **6b**, **1d**, **2d**, and **3d** are not coplanar, except for **4d**, **5d**, and **6d**. The structural distortions can be attributed to the repulsion between hydrogen atoms. In general, a diradical with good planarity is conducive to magnetic coupling. We found that the good planarity of **1c**, **2c**, **3c**, **4c**, **5c**, and **6c** leads to a larger magnetic interaction, while the magnetic coupling strength of the other diradicals has little correlation with their planarity. It is widely accepted that two shorter linking bond lengths between the coupler and two spin centers for a diradical can generate a stronger magnetic interaction [15,16]. With the elongation of the coupler, the averaged linking bond lengths of **1a** (1.374 Å), **2a** (1.383 Å), and **3a** (1.392 Å) increase gradually, and the corresponding magnetic coupling decreases successively. A similar situation occurs with **1d**, **2d**, and **3d**. On the contrary, the averaged linking bond lengths of **3b** (1.387 Å), **4b** (1.384 Å), **5b** (1.381 Å), and **6b** (1.379 Å) decrease with the extension of the coupler, and the corresponding magnetic coupling increases in sequence. The diradicals of **3c**, **4c**, **5c**, and **6c** present a similar case. It is worth mentioning that the shortest linking bond of **1d** (1.354 Å) leads to the strongest magnetic interaction (−1267.5 cm^−1^) among the studied diradicals. The linking bond lengths between the coupler and two spin centers of all NO-based diradicals shown in Figure S1 are marked in red. The geometric optimizations for the ground states of **2a′**, **2b′**, **3a′**, **3b′**, **2c′**, **2d′**, **3c′**, and **3d′** are also displayed in Figure S1. Figure 2 illustrates the redox-modulated conversions of structures for **3a ↔ 3b** and **3c ↔ 3d**.

### 2.2. Spin Polarization Analysis

To further explain the different magnetic magnitudes of the studied diradicals because of a noticeable elongation of the coupler, the spin polarization was analyzed. In general, a diradical with a shorter coupler length and an extensively π-conjugated plane structure can increase the spin density from spin center delocalization into the coupler, resulting in a larger |*J*| value. As shown in Table 1, the average spin density delocalization into the coupler decreases gradually with an increase in the coupler length for the antiferromagnetic diradicals **1d**, **2d**, **3d**, **4d**, **5d**, and **6d**, and the corresponding magnetic interaction successively weakens. Specifically, 37.0%, 23.7%, 17.8%, 16.4%, 15.3%, and 15.1% of spin densities are delocalized to the coupler for **1d**, **2d**, **3d**, **4d**, **5d**, and **6d**, corresponding to −1267.5, −589.4, −302.5, −184.4, −98.5, and −66.4 cm^−1^ in the respective order. By contrast, the average spin density delocalization into the coupler increases gradually with an extension of the coupler for the ferromagnetic diradicals **3b**, **4b**, **5b**, and **6b**, and the corresponding magnetic interaction is enhanced successively. In detail, the spin polarizations toward the coupler are 17.6%, 19.9%, 21.6%, and 22.5% for **3b**, **4b**, **5b**, and **6b**, respectively, which correspond to 408.0, 464.5, 576.6, and 740.7 cm^−1^ in sequence (Table 1). Similarly, the atomic spin density distributions in the NO radical groups are 83.1%, 80.8%, 78.7%, and 77.4% with regard to the ferromagnetic diradicals **3c**, **4c**, **5c**, and **6c**, respectively, which correspond to 339.6, 383.0, 473.3, and 621.6 cm^−1^ in turn (Table 1). A longer coupling path between two spin centers leads to a larger spin polarization, which is counterintuitive. The reason is that the elongation of the coupler reduces its HOMO-LUMO gap, which makes itself favorable to exhibit the diradical characteristic, thus promoting spin delocalization. On the whole, we can draw the conclusion that a large degree of delocalization of spin density contributes to a strong magnetic interaction, thus featuring a large |*J*| value. Apparently, for **3b**, **4b**, **5b**, and **6b**, as well as for **3c**, **4c**, **5c**, and **6c**, the increase in the percentage of spin center delocalization is not that obvious compared to the significant increase in their *J* values. In particular, a diradical with strong magnetic coupling corresponds to a small spin polarization, such as is the case for **1a** and **1b** (Table 1). These phenomena indicate that spin polarization is one of the factors affecting the magnetic interaction of diradicals but not the decisive factor. Unusually, as shown in Table 1, it is evident that the spin polarization toward the coupler has hardly changed as for **3a**, **4a**, **5a**, and **6a**. This exceptional case is related to the different spin coupling channels due to the presence of two carbonyl groups in their couplers. The Mulliken atomic spin density distributions of the twelve couples of diradicals are displayed in Figure S2.

### 2.3. Spin Coupling Channels and Spin Alternation Rule Analysis

**Figure 3:**
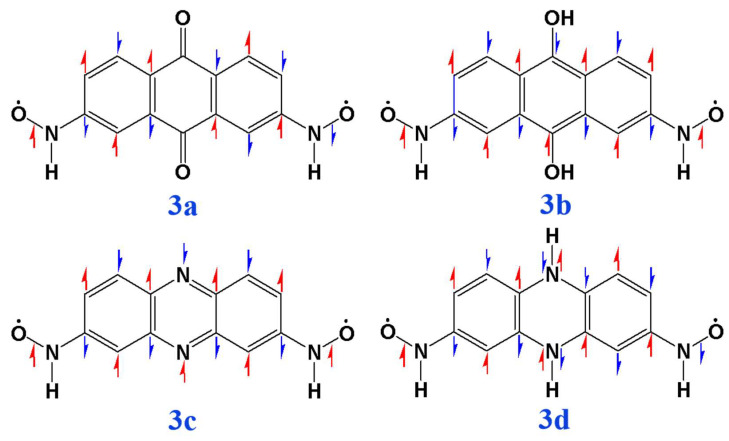
Schemes of spin alteration for **3a**, **3b**, **3c**, and **3d**. The red arrow indicates spin up and the blue arrow indicates spin down. From the figure the ferromagnetic or antiferromagnetic behavior of a diradical is visible.

As shown for the spin density distributions of the ground states of the twelve couples of diradicals in Figure 2 and Figure S3, we found that the spin coupling channels of each couple of diradicals are obviously different, which clearly demonstrates the magnetic conversion mechanism before and after dihydrogenation. For **1a**, **2a**, **3a**, **4a**, **5a**, and **6a**, the spin polarization to the coupler is hindered due to the existence of two carbonyl groups. That is, the spin-interacting pathways do not pass through the two carbonyl groups of the coupler. As a consequence, the spin transport through the coupler is blocked. In this situation, one would expect there is no magnetic interaction between two spin centers. Yet, we observed the magnetic couplings for **1a**, **2a**, **3a**, **4a**, **5a**, and **6a** to be corresponding to −74.8, −63.4, −37.9, −24.3, −15.0, and −11.2 cm^−1^ in the respective order. The superexchange pathways [42]. The intended meaning has been retained. between two spin centers in **1a**, **2a**, **3a**, **4a**, **5a**, and **6a** are dominant in magnetic coupling. That is, their magneto-structural correlations are bound to superexchange pathways [42]. In contrast, after dihydrogenation, the spin transmission through the coupler is unimpeded, and the magnetic interactions of the corresponding dihydrogenated diradicals are significantly improved. In particular, the magnetic behavior is changed from antiferromagnetism to ferromagnetism. The spin alternation rule states that the adjacent atomic center in a π-conjugated diradical prefers opposite spins: in other words, alternating α and β spins. According to this spin alternation rule, Ali and Datta reported that the sign of *J* is determined by the number of bonds in the spin-interacting pathway through the coupler [13]. If the number of bonds is odd, antiferromagnetism happens, and ferromagnetism arises when the number of bonds is even. For example, an antiferromagnetic coupling occurs for the p-phenylene-bridged diradical, while a ferromagnetic coupling is supported for the m-phenylene-bridged diradical. As depicted in Figure 2 and Figure S3, after dihydrogenation, the adjacent atomic center of **1b**, **2b**, **3b**, **4b**, **5b**, and **6b** prefers alternating α and β spins, thus following the spin alternation rule. There is an even number of bonds through their coupling pathways, showing ferromagnetism. Due to the blocking of two carbonyl groups, there is no spin on the two groups, and, thus, the adjacent atomic center of **1a**, **2a**, **3a**, **4a**, **5a**, and **6a** does not show strict alternating spin, resulting in magnetic conversion. With regard to **1c**, **2c**, **3c**, **4c**, **5c**, and **6c**, the spin transport through the coupler is unobstructed and the number of bonds in the spin-interacting pathway through their couplers is all even, corresponding to relatively large ferromagnetic couplings. After dihydrogenation, the spin transmission through the coupler is still unblocked, and we have found that every two nitrogen atoms in the coupler can provide two π-electrons, which constitute the equivalent of a chemical bond, and, thus, the spin-interaction pathways become odd in **1d**, **2d**, **3d**, **4d**, **5d**, and **6d**, leading to comparatively large antiferromagnetic interactions. Therefore, it can be concluded that the differences in the magnetic behaviors and magnetic magnitudes of each of the twelve couples of diradicals depend mainly on the visibly different spin-interacting pathways, including the spin coupling channel and the length of the coupling path discussed above. The spin alternation schemes of **3a**, **3b**, **3c**, and **3d** are shown in Figure 3, and the spin alternation schemes of the other ten couples of diradicals are shown in Figure S4.

### 2.4. SOMO Effect and SOMO-SOMO Energy Level Splitting

Furthermore, aside from the spin alternation rule, the SOMO effect and the SOMO-SOMO energy level splitting are also taken into account to predict the magnetic properties of diradicals. Borden and Davidson [38] explained that a molecule with nondisjoint (i.e., in which atoms are common) SOMOs would have a triplet ground state, while a disjoint (i.e., in which atoms are not common) shape of SOMOs in a molecule would display a singlet ground state. Moreover, the shape of SOMOs could affect the S-T energy gap of the molecule [43]. More precisely, a disjoint shape of SOMOs shows that the overlapped region of atomic orbitals occupied by two electrons is small, and, therefore, the repulsion of two electrons is accordingly weak, generating nearly degenerate ground states and, naturally, a small S-T energy gap. By contrast, in the case of a nondisjoint shape of SOMOs, the intense repulsion between two electrons in an atomic orbital supports spin-parallel orientation, leading to the triplet ground state and a large S-T energy gap. As shown in Figure S3, we found that the SOMOs of the diradicals of **1a**, **2a**, **3a**, **4a**, **5a**, **6a**, **1d**, **2d**, **3d**, **4d**, **5d**, and **6d** are disjoint in nature and are accordingly antiferromagnetic with small S-T energy gaps. Nevertheless, the diradicals of **1b**, **2b**, **3b**, **4b**, **5b**, **6b**, **1c**, **2c**, **3c**, **4c**, **5c**, and **6c** are ferromagnetic and their SOMOs are mostly nondisjoint with large S-T energy gaps. In addition, the S-T energy gap is closely related to the calculated energy level splitting of two SOMOs [43]. It seems that, in most cases, a smaller SOMO-SOMO energy level splitting (ΔE_SS_) is likely to result in a larger S-T energy gap (ΔE_ST_), and there is a linear correlation between the two.

**Figure 4:**
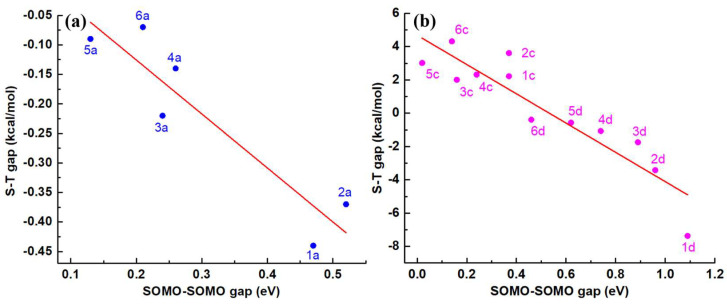
Correlation between SOMO−SOMO energy gaps of the triplet states and S−T energy gaps. (**a**) **1a**, **2a**, **3a**, **4a**, **5a**, and **6a**, (**b**) **1c**, **2c**, **3c**, **4c**, **5c**, **6c**, **1d**, **2d**, **3d**, **4d**, **5d**, and **6d**.

Hoffmann [44] proposed that if the energy difference between the two SOMOs (ΔE_SS_) is less than 1.5 eV, the two nonbonding electrons would prefer to occupy different degenerate orbitals with a parallel-spin configuration to minimize their electrostatic repulsion, thus resulting in a triplet ground state. Subsequently, Constantinides and coworkers [43] suggested that molecules with ΔE_SS_ > 1.3 eV are clear singlets via the investigation of a series of 4nπ antiaromatic linear and angular polyheteroacenes. In addition, Zhang et al. [12] indicated that the critical values of ΔE_SS_ are different for different m-phenylene-bridged diradicals. In this work, the SOMO-SOMO energy gaps of **1a**, **2a**, **3a**, **4a**, **5a**, and **6a** are relatively small (0.13–0.52 eV) but give rise to antiferromagnetic interaction. The deviation is due to the existence of two carbonyl groups in these diradicals, which inhibits the spin polarization, resulting in a strong repulsion of electrons and thus a small ΔE_SS_. As shown in Figure 4a, there is a linear relationship between ΔE_SS_ and ΔE_ST_ for **1a**, **2a**, **3a**, **4a**, **5a**, and **6a**. After dihydrogenation, the diradicals **1b**, **2b**, **3b**, **4b**, **5b**, and **6b**, with quite small ΔE_SS_ values, are known to present ferromagnetic couplings naturally, corresponding to 0.04, 0.33, 0.43, 0.49, 0.54, and 0.54 eV, respectively. With regard to **1c**, **2c**, **3c**, **4c**, **5c**, and **6c**, their ΔE_SS_ values are also very small (0.02–0.37 eV), thus resulting in ferromagnetic interaction. After dihydrogenation, the SOMO-SOMO energy gaps of **1d**, **2d**, **3d**, **4d**, **5d**, and **6d** are comparatively large (0.46–1.09 eV), leading to antiferromagnetic couplings. In particular, **1d**, **2d**, and **3d** present the singlet ground states with ΔE_SS_ values of 1.09, 0.96, and 0.89 eV, respectively, which are in close proximity to 1.3 eV. It is worth mentioning that there is a good linear relationship between ΔE_SS_ and ΔE_ST_ for **1c**, **2c**, **3c**, **4c**, **5c**, **6c**, **1d**, **2d**, **3d**, **4d**, **5d**, and **6d**, as shown in Figure 4b. Therefore, a diradical with a small ΔE_SS_ tends to have a large positive ΔE_ST_, while a diradical with a large ΔE_SS_ has a small or negative ΔE_ST_. We tabulate the energy levels of two SOMOs of the triplet state for the studied diradicals in Table S5, and their S-T energy gaps are also listed for comparison.

## 3. Computational Details

In general, both the coupler and radical groups are essential for a diradical. In this context, we theoretically designed and investigated twelve organic molecular switches, each of which consists of two nitroxide groups and a redox-property-tunable coupler. The molecular geometric optimizations, frequency analyses, and energy calculations of the closed-shell (CS) singlet, broken-symmetry (BS) open-shell singlet, and triplet (T) state of all the diradicals were carried out at the (U)B3LYP/6-311++G(d,p) level of theory. All optimized geometries were confirmed to be minimum on their potential energy surfaces, showing no imaginary frequency. For the BS state calculations, to generate the appropriate BS wave functions, we used “guess = mix” keywords. The use of “guess = mix” keywords allows the HOMO and the LUMO to be mixed, and this removes the α,β spatial symmetries and generates a new initial guess [45]. Additionally, a more modern M06-2X function with the 6-311++G(d,p) basis set was also taken into consideration to confirm the accuracy of the computational results. Moreover, to demonstrate the redox-driven magnetic conversion, the magnetic couplings of four couples of bis(tert-butyl)-nitroxide-based diradicals were also considered at the (U)B3LYP/6-311++G(d,p) level. Here, an adequately large 6-311++G(d,p) basis set with greater polarization and diffuse functions at both the B3LYP and M06-2X levels of theory was selected to obtain more reliable *J* values for the studied diradicals. An expression of the magnetic exchange coupling constant is written as *J* = (*E*_BS_ − *E*_T_)/(<*S*^2^>_T_ − <*S*^2^>_BS_), where E_BS_ and E_T_ refer to the energies of the BS and T states, respectively and <S^2^>_BS_ and <S^2^>_T_ denote the average spin square values of these spin states, respectively. This expression was developed by Yamaguchi and coworkers [46,47] and considered the most appropriate one for evaluating the *J* values. When *J* is positive, the high-spin or ferromagnetic coupling state is the ground state; when it is negative, the low-spin or antiferromagnetic coupling state is the ground state. Ginsberg [48] developed an expression of the S-T energy gap between the pure singlet state and the triplet state, and the S-T energy gap was estimated as Δ*E*_ST_ = <*S*^2^>_T_ *J*, using the abovementioned *J*, with Δ*E*_ST_ = *E*_S_ − *E*_T_. All of these DFT calculations were performed using the Gaussian 09 suite of the program [45].

## 4. Conclusions

In conclusion, we theoretically designed twelve couples of NO-based diradicals, each of which can undergo the magnetic transformation between ferromagnetism and antiferromagnetism via redox induction (**1a ↔ 1b**, **2a ↔ 2b**, **3a ↔ 3b**, **4a ↔ 4b**, **5a ↔ 5b**, **6a ↔ 6b**, **1c ↔ 1d**, **2c ↔ 2d**, **3c ↔ 3d**, **4c ↔ 4d**, **5c ↔ 5d**, and **6c ↔ 6d**). The computational results reveal that the antiferromagnetic interactions of **1a**, **2a**, **3a**, **4a**, **5a**, and **6a** decrease with a noticeable elongation of the coupler. In particular, the presence of two carbonyl groups in their couplers hampers spin polarization and weakens the magnetic coupling between two spin centers greatly, resulting in small |*J*| values. Their magneto-structural correlations are bound to superexchange pathways. After dihydrogenation, the ferromagnetic couplings of **1b**, **2b**, **3b**, **4b**, **5b**, and **6b** decrease first and then increase with the extension of the coupler. The shorter coupler lengths and linking bond lengths of **1b** and **2b** and the smaller HOMO-LUMO gap of the coupler, as well as the larger spin polarization for **3b**, **4b**, **5b**, and **6b**, are critical in enhancing their corresponding magnetic couplings successively. The magnetic conversion of these six couples of diradicals before and after dihydrogenation is attributed to the different spin coupling channels. Before dihydrogenation, the spin transmission through the coupler is blocked due to the presence of two carbonyl groups, but, after dihydrogenation, the spin transport follows the spin alternation rule, resulting in magnetic switching.

Similarly, the ferromagnetic interactions of **1c**, **2c**, **3c**, **4c**, **5c**, and **6c** decrease first and then increase as the length of the coupler extends. The shorter coupler lengths and linking bond lengths of **1c** and **2c** and the smaller HOMO-LUMO gap of the coupler, as well as the larger spin polarization for **3c**, **4c**, **5c**, and **6c**, are crucial in promoting their corresponding magnetic couplings progressively. After dihydrogenation, the antiferromagnetic couplings of **1d**, **2d**, **3d**, **4d**, **5d**, and **6d** gradually weaken as the length of the coupler increases. The spin polarization plays a key role in controlling their magnetic couplings, and their corresponding average spin density delocalization into the coupler decreases in sequence. The magnetic transformation of these six couples of diradicals occurs due to the number of bonds in the spin-interacting pathway through the coupler changing from even to odd after dihydrogenation.

The ground states of the twelve couples of diradicals can be explained on the basis of the spin alternation rule, the SOMO effect, and the SOMO-SOMO energy gaps of the triplet states. In the spin-trapped solution matrix, **1b**, **2b**, **3b**, **4b**, **5b**, **6b**, **1c**, **2c**, **3c**, **4c**, **5c**, and **6c** could store magnetic information as an “ON” state at the high-spin ground state, while **1a**, **2a**, **3a**, **4a**, **5a**, **6a**, **1d**, **2d**, **3d**, **4d**, **5d** and **6d**, could store information as an “OFF” state in the low-spin ground state. We believe that this work would open a new prospect for the rational design of such redox-regulated diradicals and find further technical applications in magnetic molecular switches. Of course, we still need to make great attempts to explore the magnetic conversion mechanisms through the redox process in a real system.

## Figures and Tables

**Table 1:** Average spin density delocalization into coupler (%) and corresponding intramolecular magnetic coupling constants (*J*, in cm^−1^) for all studied nitroxide-based diradicals.

Species	Average Spin Density Delocalization into Coupler (%)	*J*	Species	Average Spin Density Delocalization into Coupler (%)	*J*
**1a**	25.7	−74.8	**1b**	13.8	551.6
**2a**	20.1	−63.4	**2b**	16	417.2
**3a**	15.1	−37.9	**3b**	17.6	408
**4a**	15	−24.3	**4b**	19.9	464.5
**5a**	14.9	−15.0	**5b**	21.6	576.6
**6a**	15.3	−11.2	**6b**	22.5	740.7
**1c**	13.6	621.7	**1d**	37	−1267.5
**2c**	14.7	380.9	**2d**	23.7	−589.4
**3c**	16.9	339.6	**3d**	17.8	−302.5
**4c**	19.2	383	**4d**	16.4	−184.4
**5c**	21.3	473.3	**5d**	15.3	−98.5
**6c**	22.6	621.6	**6d**	15.1	−66.4

## Data Availability

The data that support the findings of this study are available from the corresponding author upon reasonable request.

## Supplementary Materials

The following supporting information can be downloaded at https://www.mdpi.com/article/10.3390/molecules28176232/s1: All estimated data and figures calculated at the (U)B3LYP/6-311++G (d,p) level, including the energies for the close-shell singlet, broken-symmetry open-shell singlet and triplet, <S^2^> values, and intramolecular magnetic exchange coupling constants, as well as singlet–triplet energy gaps for all designed diradicals; HOMO and LUMO energy levels as well as HOMO-LUMO energy gaps of couplers; SOMO energies and SOMO-SOMO energy gaps of triplet states; optimized geometries and bond lengths of adjacent atoms between two spin centers; Mulliken atomic spin density distributions; SOMOs and spin density maps; spin alternation plots.
