# Supplementary material for: Redox-Regulated Magnetic Conversions between Ferro- and Antiferromagnetism in Organic Nitroxide Diradicals
Source: Molecules. 2023 Aug 24;28(17):6232. doi: 10.3390/molecules28176232 (PMC10488413; doi:10.3390/molecules28176232)
Supplement: Supplementary file 1 [file molecules-28-06232-s001.zip › molecules-2536791-supplementary.pdf]

Supporting Information

Fengying Zhang [1,2,*], Zijun Zhang [3], Yali Zhao [1,2,*], Chao Du [1,2], Yong Li [1,2], Jiaqi Gao [1,2], Xiaobo Ren [1,2], Teng Ma [1,2], Boqiong Li [1,2] and Yuxiang Bu [4]

[1] Department of Materials Science and Engineering, Jinzhong University, Jinzhong 030619, China; zx88dc@sina.com (C.D.); iytyut@126.com (Y.L.); gjq5207@outlook.com (J.G.); rxb@jzxy.edu.cn (X.R.);
mateng1022@tyust.edu.cn (T.M.); lbq@jzxy.edu.cn (B.L.)
[2] Shanxi Province Collaborative Innovation Center for Light Materials Modification and Application,
Jinzhong 030619, China
[3] Department of Chemistry and Chemical Engineering, Jinzhong University, Jinzhong 030619, China; 18334858404@163.com
[4] School of Chemistry and Chemical Engineering, Shandong University, Jinan 250100, China; byx@sdu.edu.cn
* Correspondence: zfy13233005848@163.com (F.Z.); yaliz12@163.com (Y.Z.)

1. Relevant Data of Describing Ground States for All Designed Diradicals, Their Diradical Character and Magnetic Behaviors

Table S1. (U)B3LYP/6-311++G(d,p) level estimated energies (in a.u.) of the closed-shell (CS) singlet, broken-symmetry (BS) open-shell singlet and triplet (T) state, corresponding $<S^2>$ values, intramolecular magnetic coupling constants (J, in cm^{-1}) as well as S-T energy gaps (ΔE_{ST}, kcal/mol) for all studied nitroxide-based diradicals

Species	$E_{(CS)}$	$E_{(BS)}$ ($<S^2>$)	$E_{(T)}$ ($<S^2>$)	J	ΔE_{ST}
**1a**	-641.4380263	-641.4634223(1.034)	-641.4630648(2.082)	-74.8	-0.44
**1b**	-642.6473530	-642.6867238(1.005)	-642.6893172(2.036)	551.6	3.21
**2a**	-795.1248350	-795.1560298(1.035)	-795.1557353(2.054)	-63.4	-0.37
**2b**	-796.3303225	-796.3700529(1.014)	-796.3720239(2.050)	417.2	2.44
**3a**	-948.8084098	-948.8452382(1.026)	-948.8450641(2.034)	-37.9	-0.22
**3b**	-950.0009064	-950.0400077(1.022)	-950.0419894(2.087)	408.0	2.43
**4a**	-1102.4838078	-1102.5234243(1.031)	-1102.5233129(2.036)	-24.3	-0.14
**4b**	-1103.6719639	-1103.7118323(1.038)	-1103.7142024(2.157)	464.5	2.86
**5a**	-1256.1593704	-1256.2012445(1.036)	-1256.2011761(2.038)	-15.0	-0.09
**5b**	-1257.3416378	-1257.3807977(1.055)	-1257.3840817(2.304)	576.6	3.79
**6a**	-1409.8314648	-1409.8740725(1.050)	-1409.8740212(2.051)	-11.2	-0.07
**6b**	-1411.0054185	-1411.0494578(1.146)	-1411.0540885(2.517)	740.7	5.33
**1c**	-524.2320891	-524.2848499(1.009)	-524.2877531(2.033)	621.7	3.61
**1d**	-525.4602392	-525.4651502(0.768)	-525.4578212(2.036)	-1267.5	-7.37
**2c**	-677.9224919	-677.9653040(1.014)	-677.9670894(2.042)	380.9	2.22
**2d**	-679.1392148	-679.1531832(0.974)	-679.1503393(2.032)	-589.4	-3.43
**3c**	-831.5986800	-831.6375954(1.023)	-831.6392154(2.069)	339.6	2.01

**3d**	-832.8271046	-832.8500366(1.015)	-832.8486393(2.028)	-302.5	-1.75
**4c**	-985.2703056	-985.3081681(1.036)	-985.3100701(2.125)	383.0	2.32
**4d**	-985.5017878	-986.5314217(1.027)	-986.5305793(2.029)	-184.4	-1.07
**5c**	-1138.9406872	-1138.9770855(1.059)	-1138.9796257(2.236)	473.3	3.02
**5d**	-1140.1774835	-1140.2125906(1.033)	-1140.2121423(2.031)	-98.5	-0.57
**6c**	-1292.6097857	-1292.6453885(1.096)	-1292.6491806(2.434)	621.6	4.32
**6d**	-1293.8491436	-1293.8867316(1.045)	-1293.8864305(2.039)	-66.4	-0.39

Table S2. (U)M06-2X/6-311++G(d,p) level estimated energies (in a.u.) of the broken-symmetry (BS) open-shell singlet and triplet (T) state, corresponding <S^2> values, intramolecular magnetic coupling constants (*J*, in cm^{-1}) for all studied nitroxide-based diradicals

Species	$E_{(BS)}$ (<S^2>)	$E_{(T)}$ (<S^2>)	*J*
**1a**	-641.2044049(1.065)	-641.2043554(2.089)	-10.6
**1b**	-642.4329108(1.010)	-642.4348843(2.034)	422.6
**2a**	-794.8393614(1.048)	-794.8393233(2.057)	-8.5
**2b**	-796.0555291(1.019)	-796.0569467(2.046)	302.7
**3a**	-948.4710141(1.030)	-948.4709925(2.034)	-4.7
**3b**	-949.6639157(1.026)	-949.6652353(2.073)	276.4
**4a**	-1102.0882483(1.032)	-1102.0882310(2.034)	-3.8
**4b**	-1103.2734185(1.039)	-1103.2748595(2.123)	291.5
**5a**	-1255.7050989(1.034)	-1255.7050882(2.035)	-2.3
**5b**	-1256.8795817(1.052)	-1256.8815429(2.246)	360.2
**6a**	-1409.3160678(1.042)	-1409.3160602(2.043)	-1.7
**6b**	-1410.4853052(1.090)	-1410.4883008(2.483)	471.6
**1c**	-524.0739089(1.012)	-524.0763106(2.034)	515.3
**1d**	-525.2441507(0.994)	-525.2406114(2.039)	-742.7
**2c**	-677.6928119(1.018)	-677.6941157(2.039)	280.0
**2d**	-678.8748725(1.033)	-678.8736934(2.034)	-258.3
**3c**	-831.3019635(1.027)	-831.3030659(2.060)	234.0
**3d**	-832.5121497(1.033)	-832.5116849(2.029)	-102.3
**4c**	-984.9101606(1.039)	-984.9113111(2.097)	238.5
**4d**	-986.1328909(1.033)	-986.1325996(2.029)	-64.1
**5c**	-1138.5159686(1.056)	-1138.5174119(2.176)	282.6
**5d**	-1139.7535376(1.033)	-1139.7533716(2.030)	-36.5
**6c**	-1292.1212049(1.080)	-1292.1233559(2.352)	370.8

**6d**	-1293.3659531(1.040)	-1293.3658228(2.036)	-28.7

Table S3. (U)B3LYP/6-311++G(d,p) level estimated energies (in a.u.) of the broken-symmetry (BS) open-shell singlet and triplet (T) state, corresponding $\langle S^2 \rangle$ values, intramolecular magnetic coupling constants (*J*, in cm^{-1}) for four couples of bis(tert-butyl)-nitroxide-based diradicals

**Species**	$E_{(BS)}$ ($\langle S^2 \rangle$)	$E_{(T)}$ ($\langle S^2 \rangle$)	*J*
**2a′**	-1109.7289295(1.023)	-1109.7285674(2.041)	-78.0
**2b′**	-1110.9444029(1.014)	-1110.9449151(2.025)	111.1
**3a′**	-1263.4271834(1.022)	-1263.4269754(2.030)	-45.2
**3b′**	-1264.6185796(1.021)	-1264.6201755(2.074)	332.3
**2c′**	-992.5502641(1.012)	-992.5516567(2.036)	298.2
**2d′**	-993.7322030(0.990)	-993.7301652(2.028)	-430.5
**3c′**	-1146.2180983(1.019)	-1146.2194055(2.059)	275.6
**3d′**	-1147.4265221(1.019)	-1147.4255500(2.024)	-212.1

2. HOMO and LUMO Energy Levels and HOMO-LUMO Gaps of Couplers for Ferromagnetic Diradicals

Table S4. The HOMO and LUMO energy levels (in a.u.) as well as HOMO-LUMO energy gaps (in eV) of the couplers for **1b**, **2b**, **3b**, **4b**, **5b**, **6b**, **1c**, **2c**, **3c**, **4c**, **5c** and **6c**

**Species**	HOMO	LUMO	HOMO-LUMO gap
**1b**	-0.21624	-0.02532	5.19
**2b**	-0.20364	-0.04993	4.18

**3b**	-0.18824	-0.07304	3.13
**4b**	-0.17981	-0.08907	2.47
**5b**	-0.17194	-0.09962	1.97
**6b**	-0.16691	-0.10785	1.61
**1c**	-0.26357	-0.06933	5.29
**2c**	-0.25900	-0.08638	4.70
**3c**	-0.23666	-0.10346	3.62
**4c**	-0.21393	-0.11366	2.73
**5c**	-0.20275	-0.12250	2.18
**6c**	-0.19179	-0.12798	1.74

3. SOMO Energies, SOMO-SOMO Energy Gaps of Triplet States and S-T Energy Gaps of All Diradicals

Table S5. The SOMO energies (a.u.) of the triplet states, SOMO-SOMO energy gaps (ΔE_{SS} in eV) and singlet-triplet energy gaps (ΔE_{ST} in kcal/mol) of **1a**, **1b**, **2a**, **2b**, **3a**, **3b**, **4a**, **4b**, **5a**, **5b**, **6a**, **6b**, **1c**, **1d**, **2c**, **2d**, **3c**, **3d**, **4c**, **4d**, **5c**, **5d**, **6c** and **6d**

Species	^1E_S	^2E_S	ΔE_{SS}	ΔE_{ST}
**1a**	-0.26895	-0.25176	0.47	-0.44
**1b**	-0.22440	-0.22309	0.04	3.21
**2a**	-0.25497	-0.23599	0.52	-0.37
**2b**	-0.21725	-0.20529	0.33	2.44
**3a**	-0.23748	-0.22857	0.24	-0.22
**3b**	-0.21282	-0.19715	0.43	2.43
**4a**	-0.23136	-0.22176	0.26	-0.14
**4b**	-0.20879	-0.19062	0.49	2.86
**5a**	-0.22320	-0.21825	0.13	-0.09
**5b**	-0.20630	-0.18635	0.54	3.79
**6a**	-0.21973	-0.21214	0.21	-0.07
**6b**	-0.20370	-0.18369	0.54	5.33
**1c**	-0.25389	-0.24039	0.37	3.61
**1d**	-0.22052	-0.18064	1.09	-7.37
**2c**	-0.23978	-0.22624	0.37	2.22
**2d**	-0.21425	-0.17891	0.96	-3.43
**3c**	-0.22591	-0.21998	0.16	2.01
**3d**	-0.20933	-0.17656	0.89	-1.75
**4c**	-0.20749	-0.19857	0.24	2.32
**4d**	-0.20479	-0.17773	0.74	-1.07

**5c**	-0.21143	-0.21064	0.02	3.02
**5d**	-0.20318	-0.18038	0.62	-0.57
**6c**	-0.20983	-0.20463	0.14	4.32
**6d**	-0.19871	-0.18186	0.46	-0.39

4. Optimized Molecular Geometries for Ground States of All Diradicals

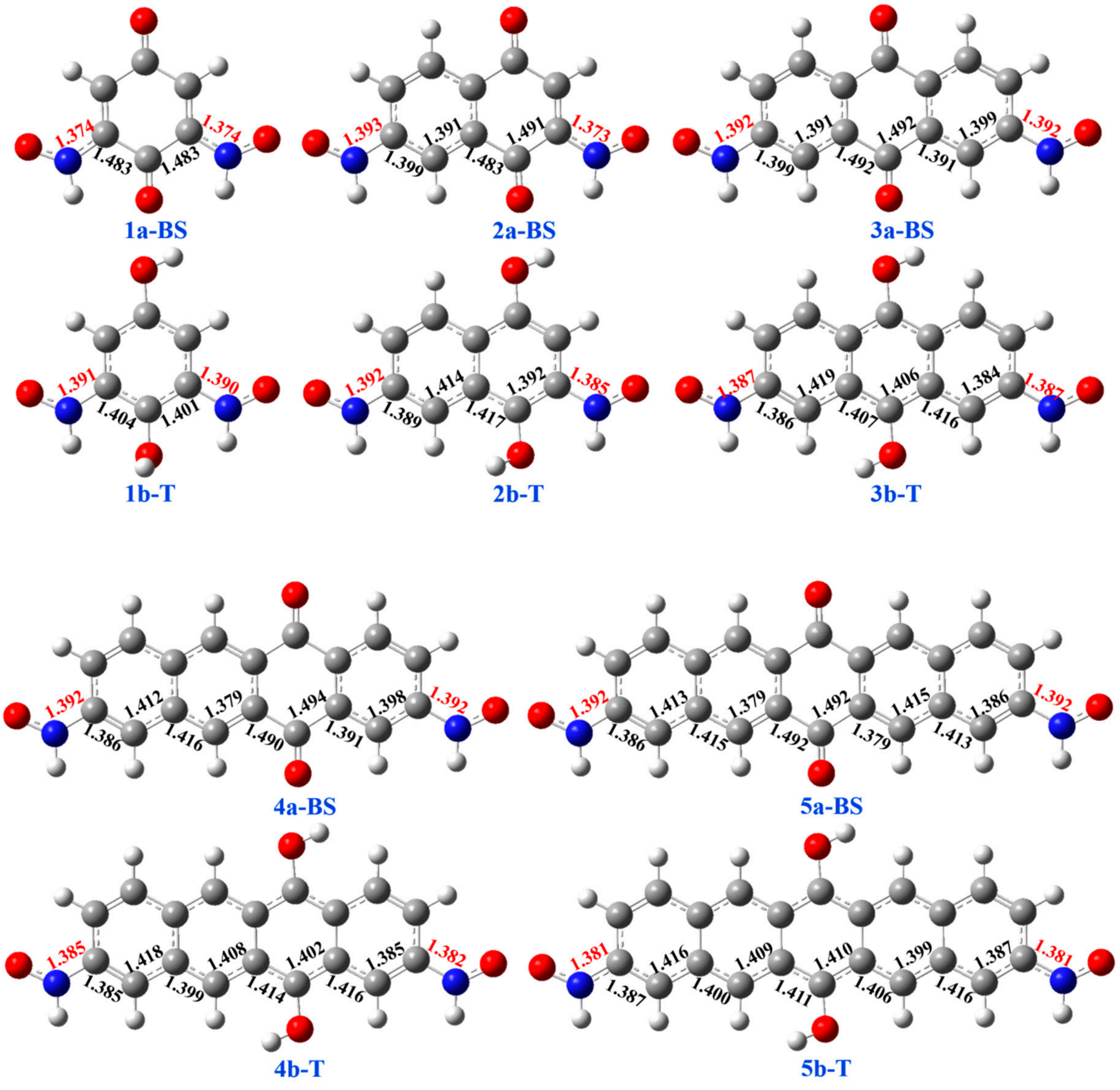

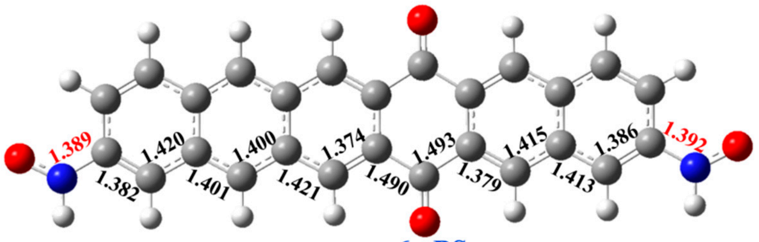

6a-BS

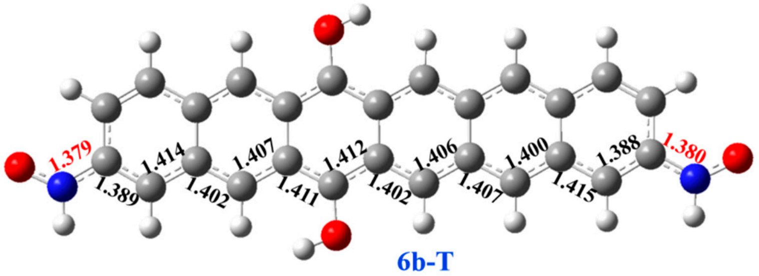

6b-T

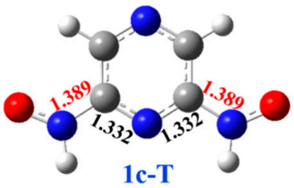

1c-T

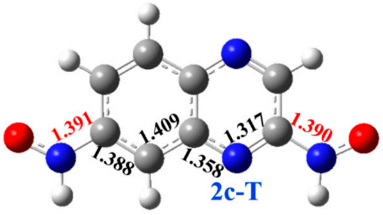

2c-T

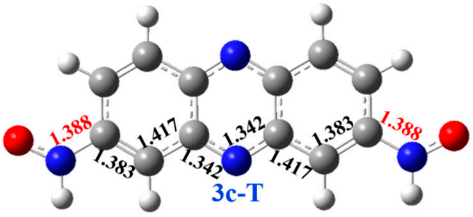

3c-T

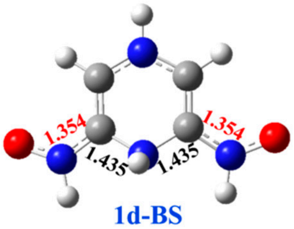

1d-BS

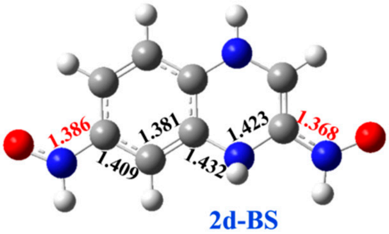

2d-BS

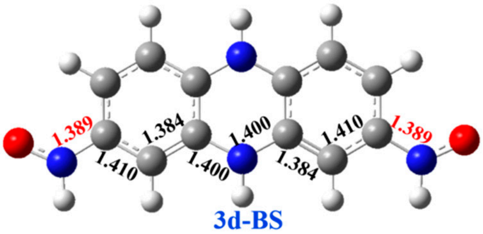

3d-BS

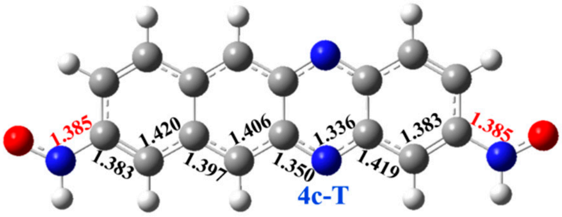

4c-T

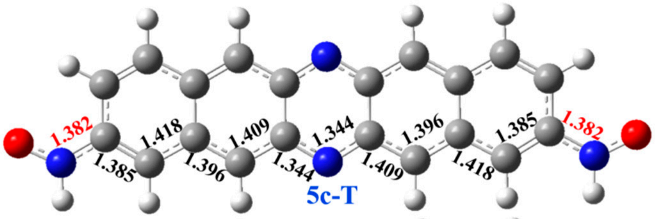

5c-T

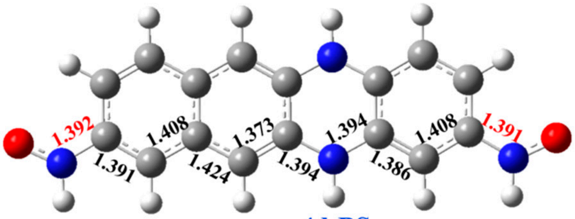

4d-BS

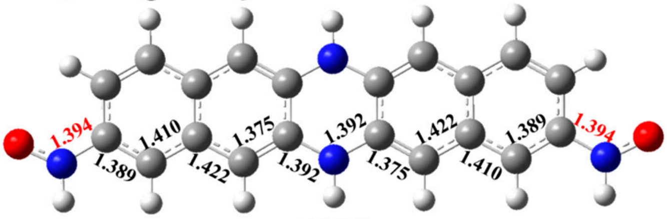

5d-BS

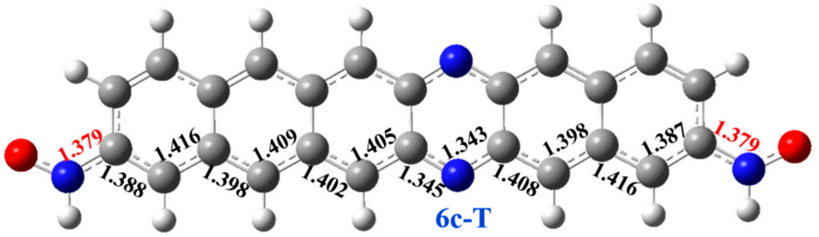

6c-T

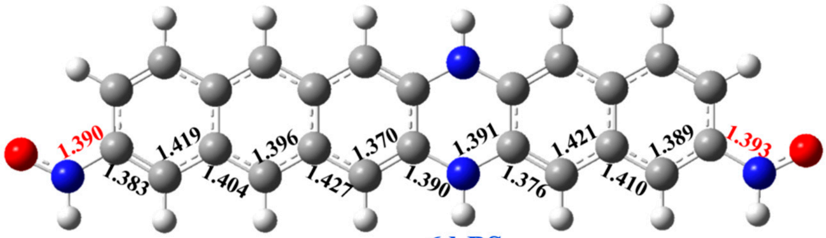

6d-BS

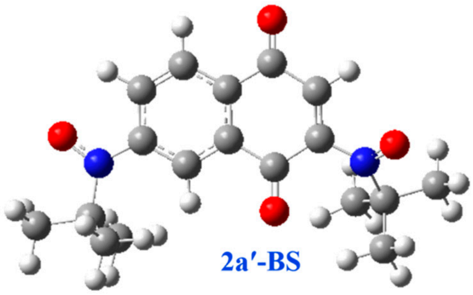

2a′-BS

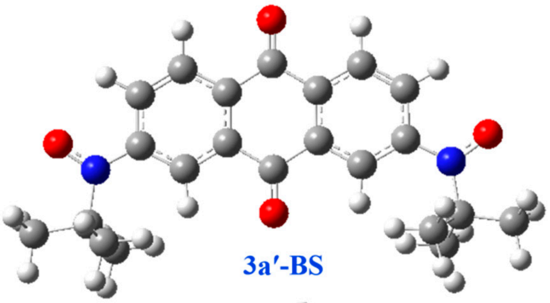

3a′-BS

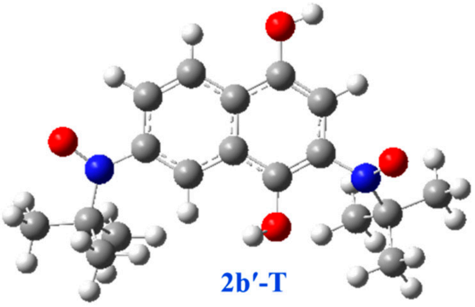

2b′-T

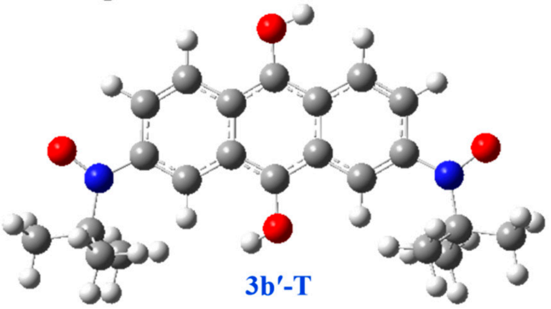

3b′-T

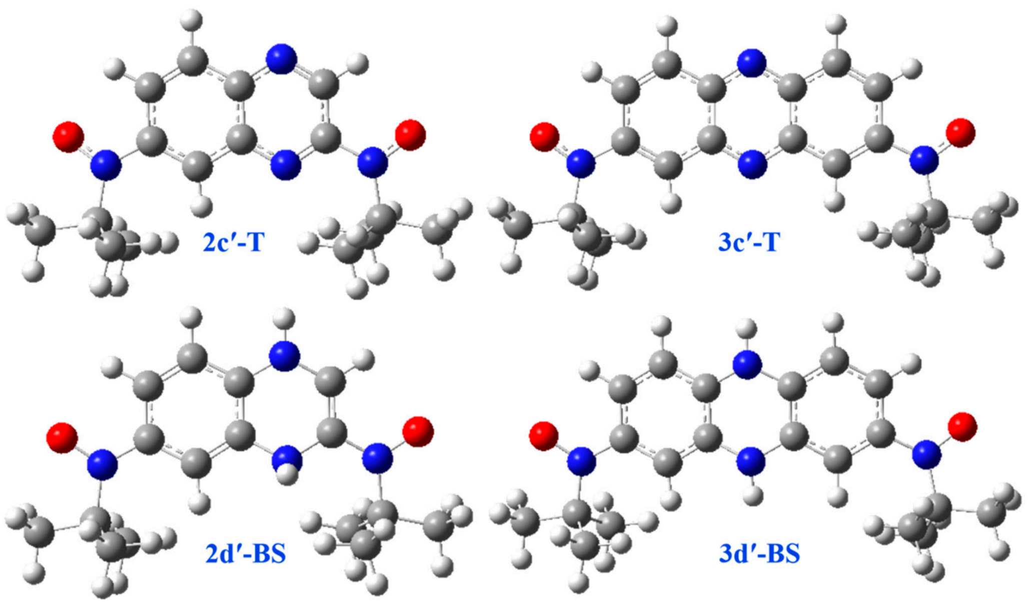

Figure S1. Optimized geometries for the ground states of all diraidcals at the (U)B3LYP/6-311++G (d,p) level, including the bond lengths of the adjacent atoms between two spin centers of the nitroxide-based diradicals. Their linking bond lengths between the coupler and two spin centers are marked in red.

5. Distributions of Mulliken Atomic Spin Density of All Diradicals

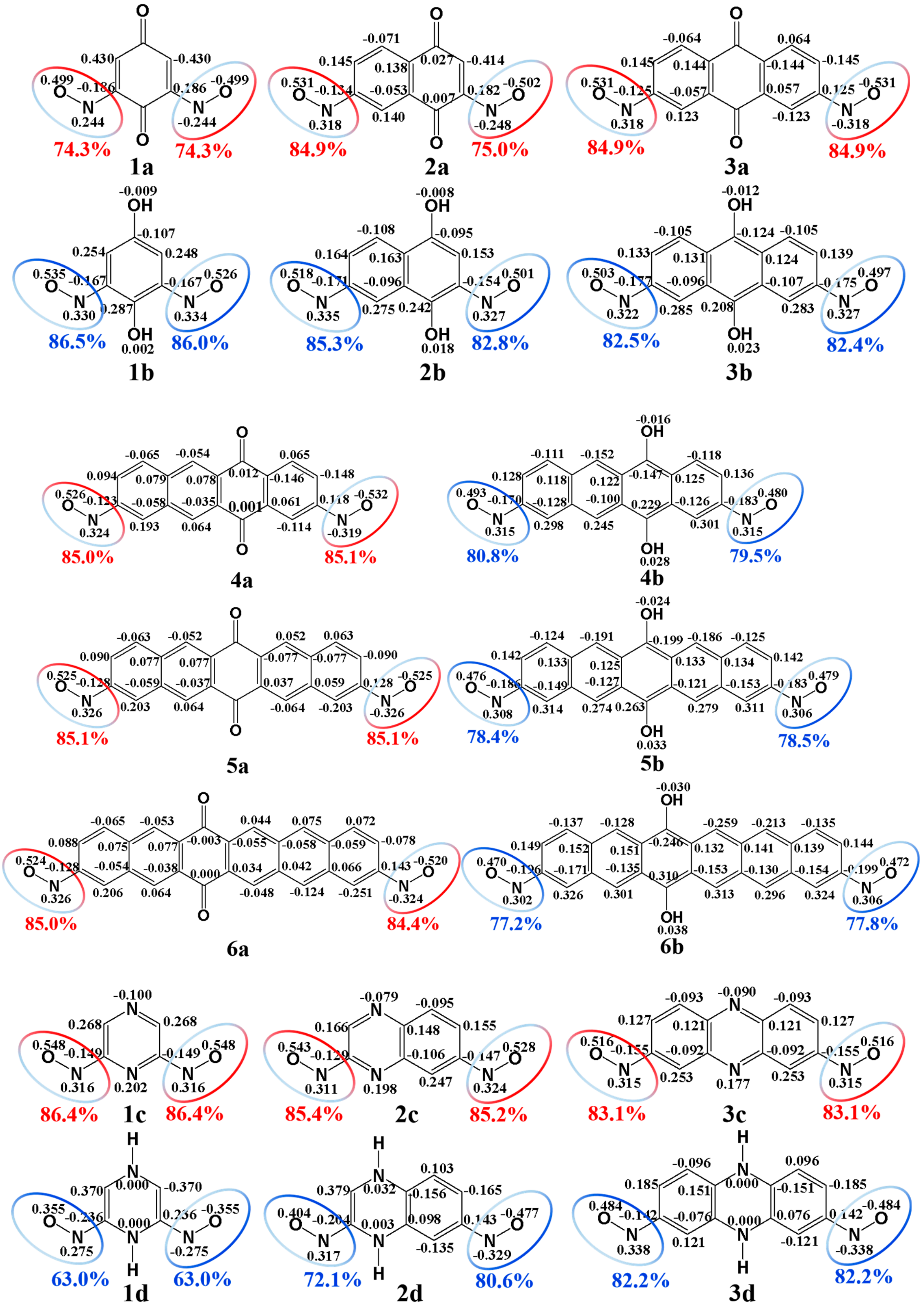
74.3%
74.3%
1a
84.9%
75.0%
2a
84.9%
84.9%
3a
86.5%
86.0%
1b
85.3%
82.8%
2b
82.5%
82.4%
3b
85.0%
85.1%
4a
80.8%
79.5%
4b
85.1%
85.1%
5a
78.4%
78.5%
5b
85.0%
84.4%
6a
77.2%
77.8%
6b
86.4%
86.4%
1c
85.4%
85.2%
2c
83.1%
83.1%
3c
63.0%
63.0%
1d
72.1%
80.6%
2d
82.2%
82.2%
3d

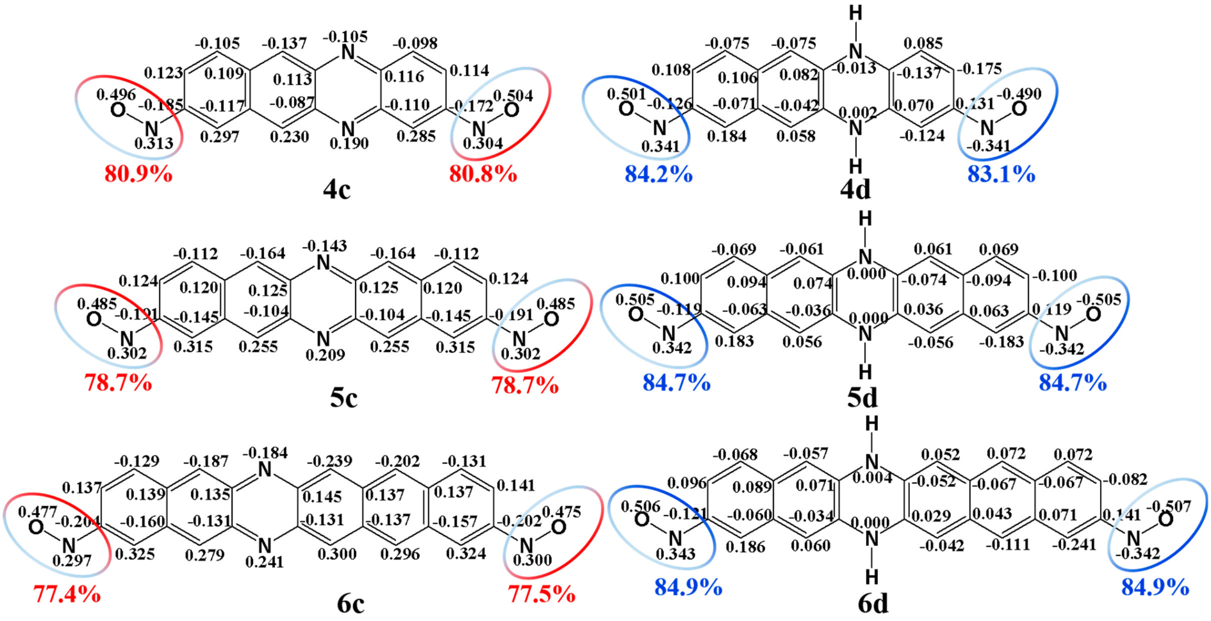

Figure S2. Comparison of spin polarization and the Mulliken atomic spin density distributions of all diradicals before and after dihydrogenation.

6. SOMO Plots and Spin Density Maps for All Diradicals

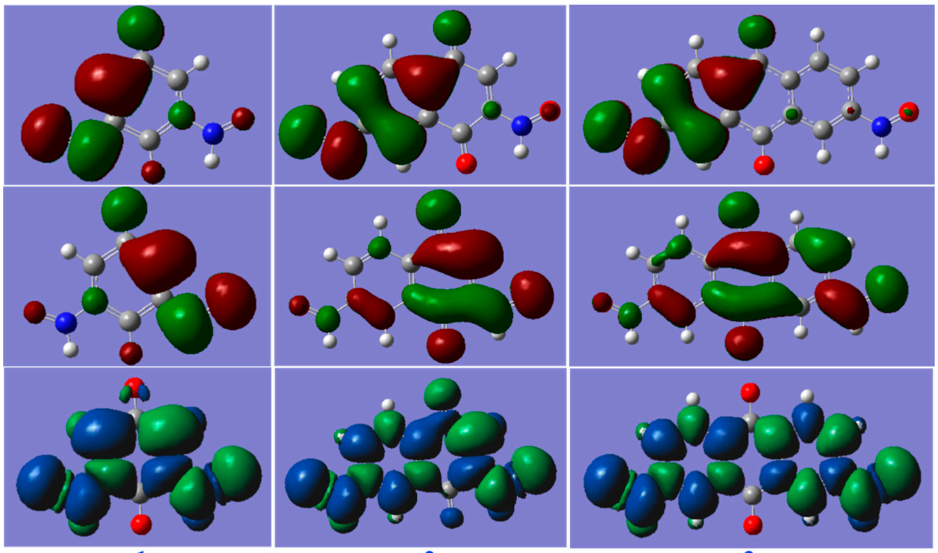

1a 2a 3a

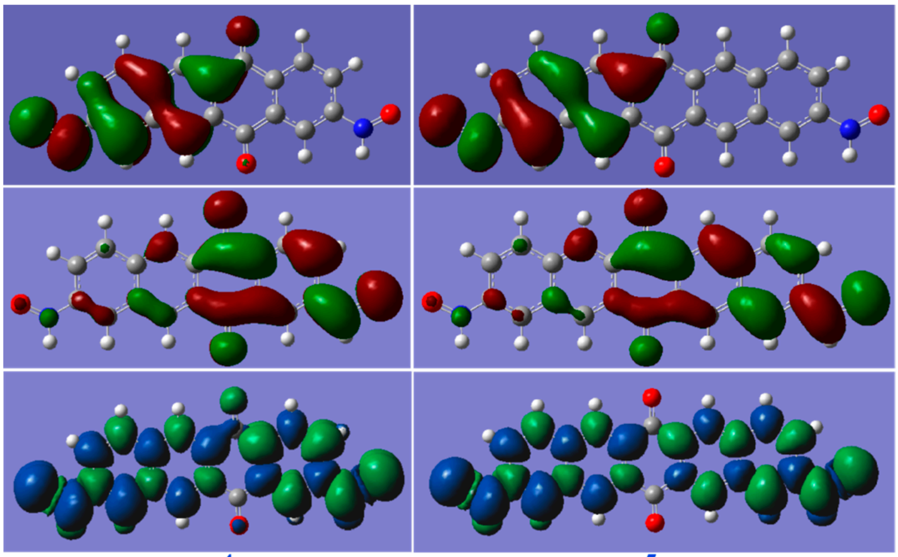

4a **5a**

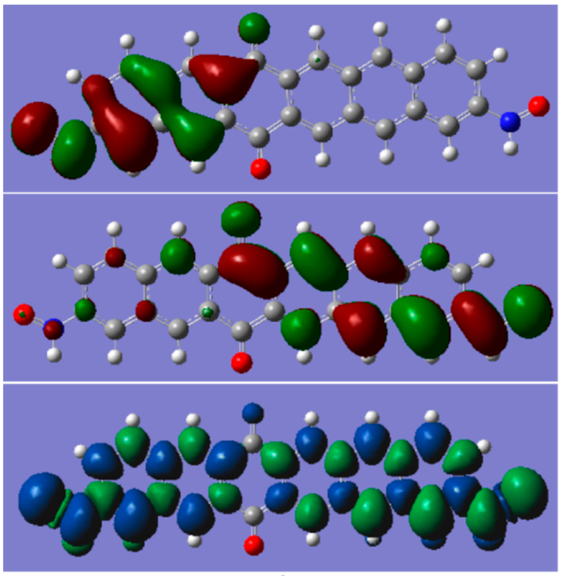

6a

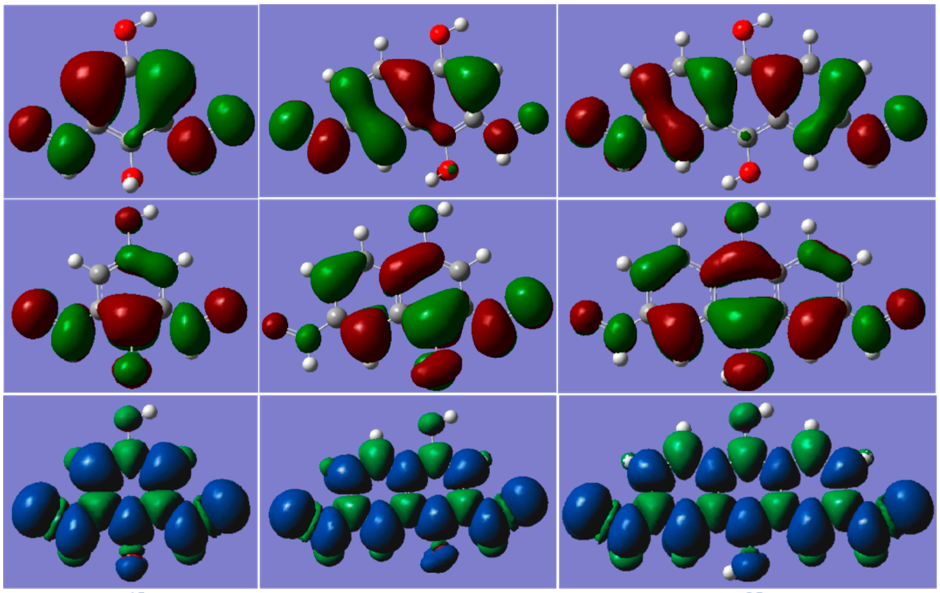

1b **2b** **3b**

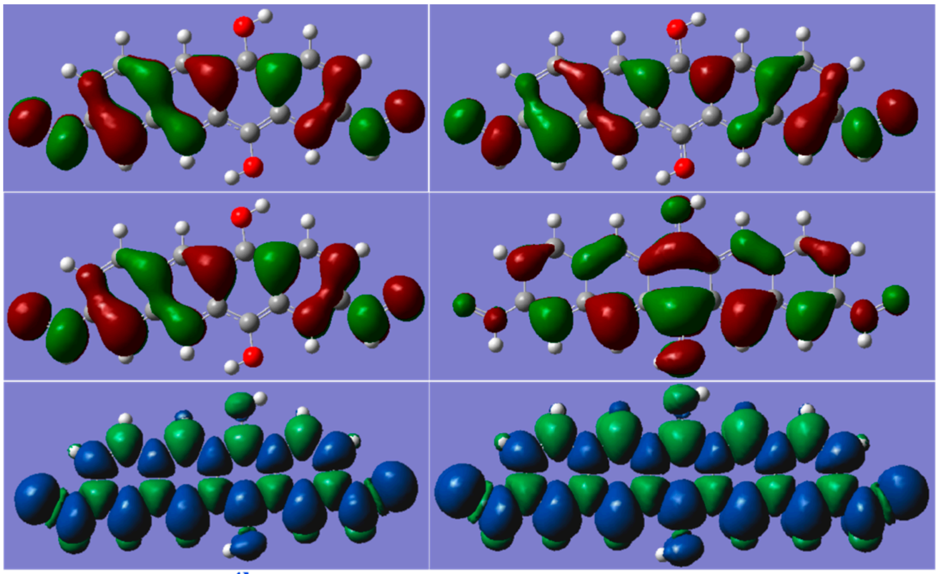

4b **5b**

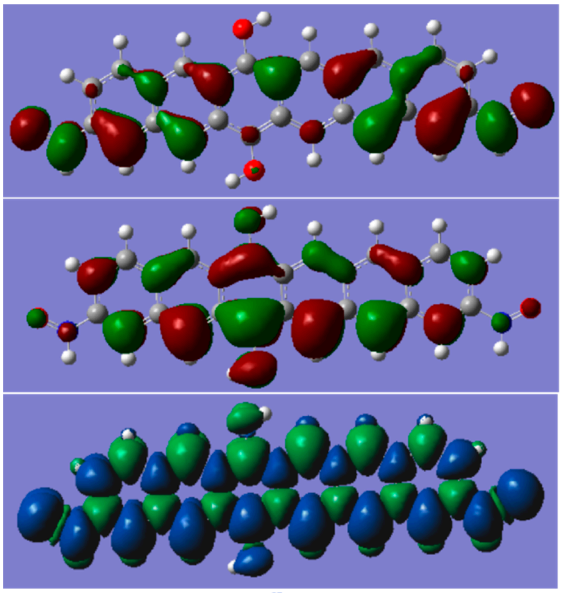

6b

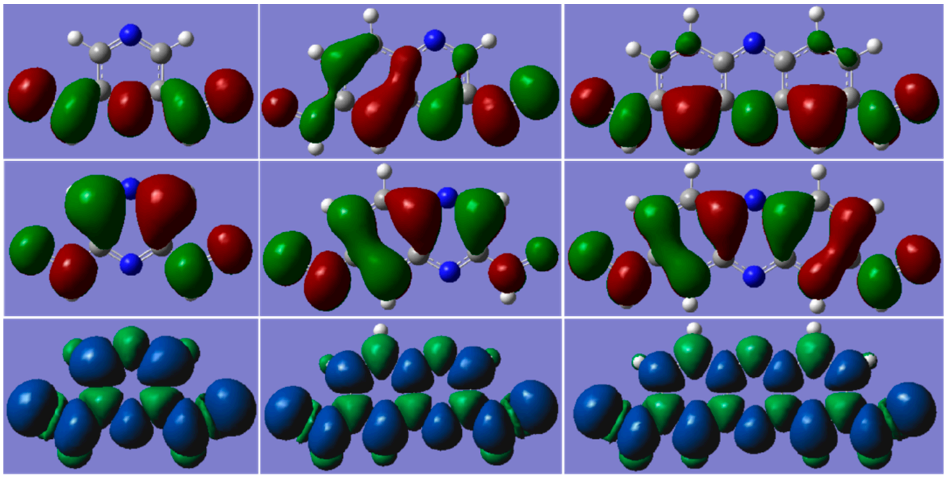

1c **2c** **3c**

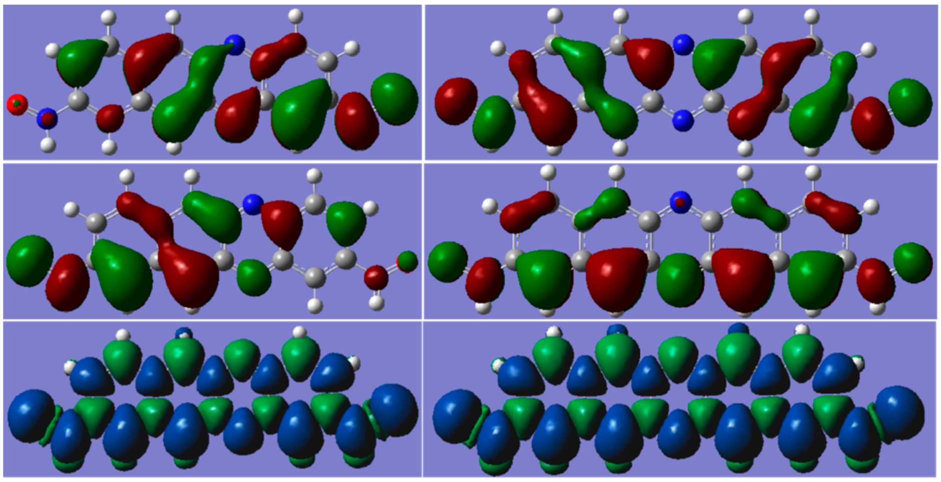

4c **5c**

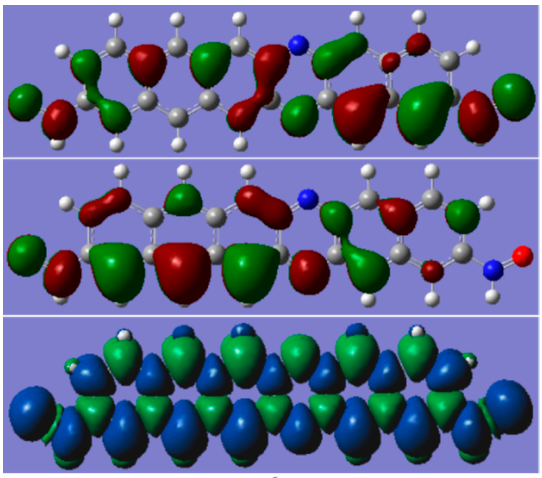

6c

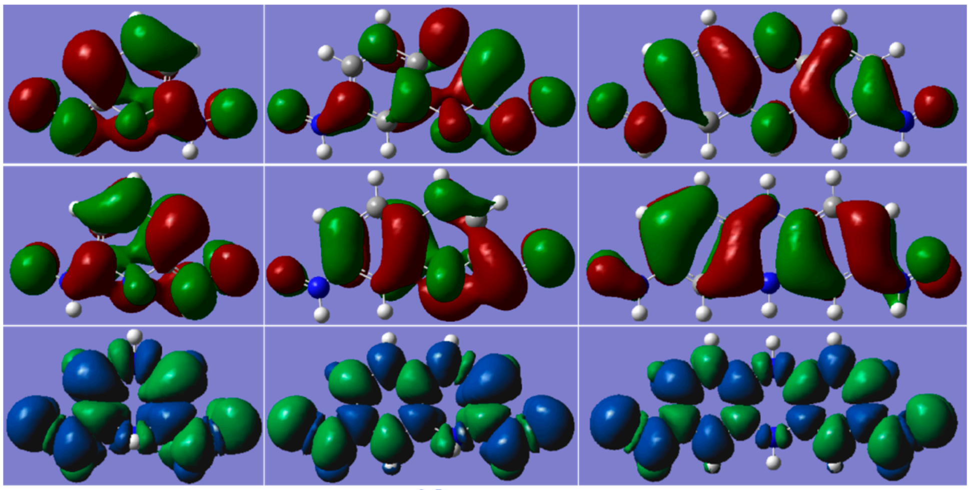

1d **2d** **3d**

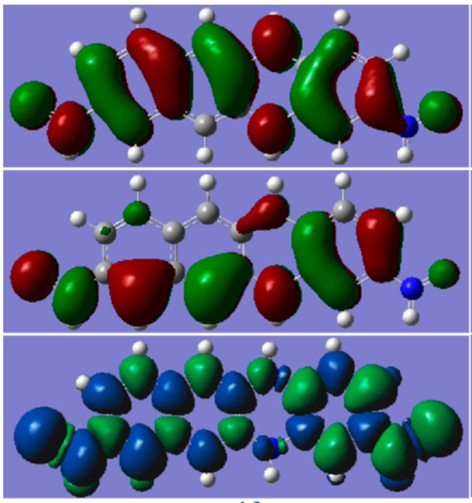

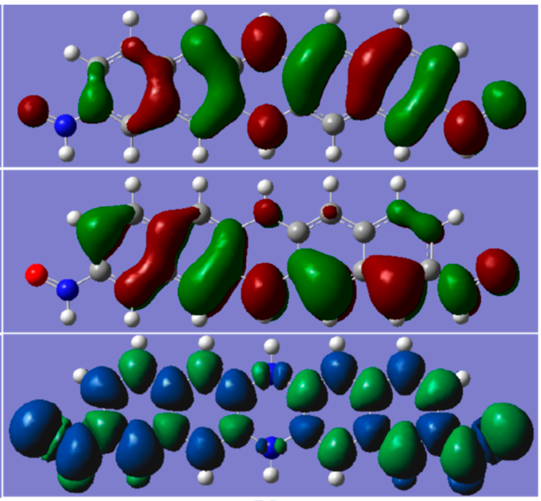

4d **5d**

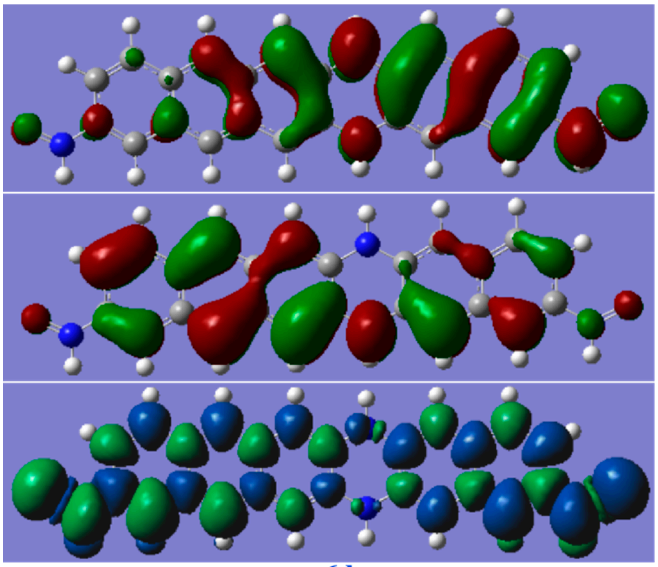

6d

Figure S3. SOMOs (isovalue = 0.02) and spin density map (isovalue = 0.0004) of the ground states for the diradicals **1a**, **2a**, **3a**, **4a**, **5a**, **6a**, **1b**, **2b**, **3b**, **4b**, **5b**, **6b**, **1c**, **2c**, **3c**, **4c**, **5c**, **6c**, **1d**, **2d**, **3d**, **4d**, **5d** and **6d**.

7. Spin Alteration Plots for for Ten Couples of Diradicals

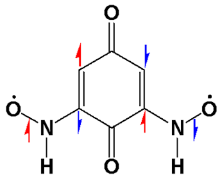

1a

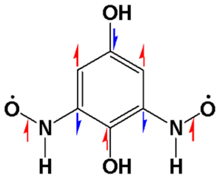

1b

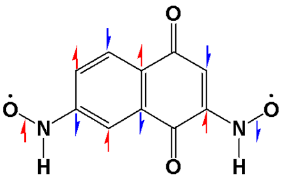

2a

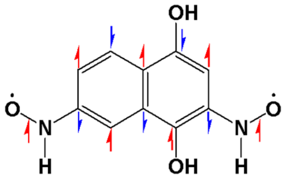

2b

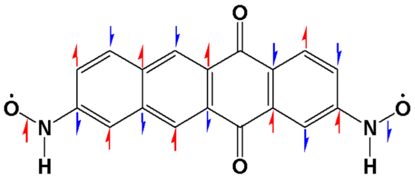

4a

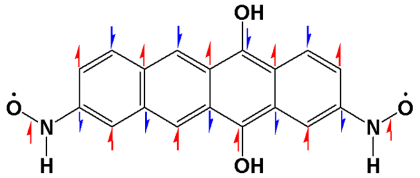

4b

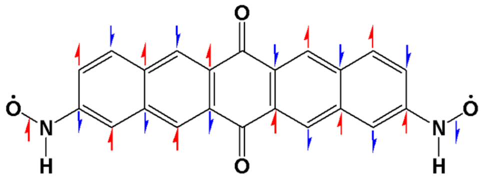

5a

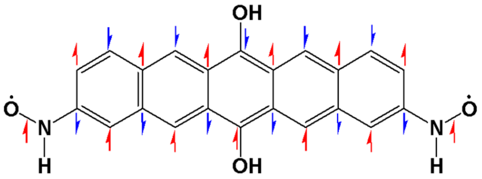

5b

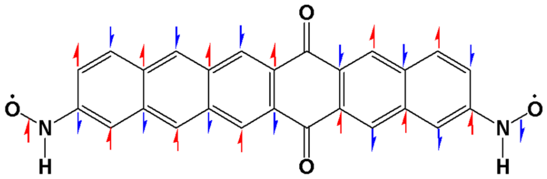

6a

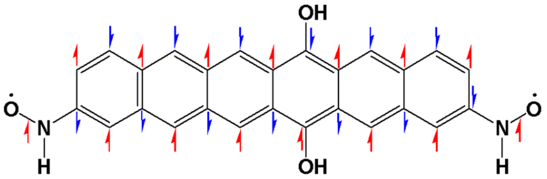

6b

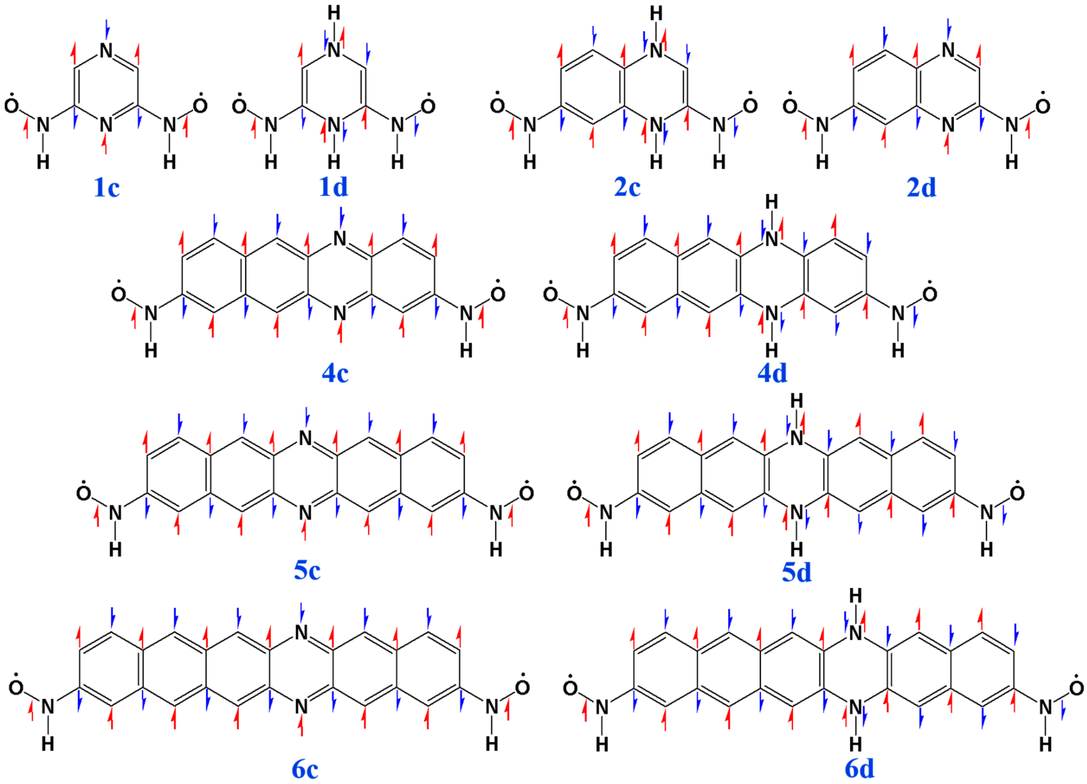

Figure S4. Scheme of spin alteration rule for ten couples of diradicals, including **1a** ↔ **1b**, **2a** ↔ **2b**, **4a** ↔ **4b**, **5a** ↔ **5b**, **6a** ↔ **6b**, **1c** ↔ **1d**, **2c** ↔ **2d**, **4c** ↔ **4d**, **5c** ↔ **5d** and **6c** ↔ **6d**.
